# 1-Naphthyl quinoxalin-2-yl ether

**DOI:** 10.1107/S1600536809007867

**Published:** 2009-03-11

**Authors:** Noor Doha Hassan, Hairul Anuar Tajuddin, Zanariah Abdullah, Seik Weng Ng

**Affiliations:** a Department of Chemistry, University of Malaya, 50603 Kuala Lumpur, Malaysia

## Abstract

In the crystal structure of the title compound, C_18_H_12_N_2_O, the dihedral angle between the two fused-ring systems is 84.3 (1) °; the C—O—C angle at the ether O atom is 117.31 (18)°.

## Related literature

For the crystal structure of the two forms of quinoxalinyl 2-phenyl ether, see: Abdullah & Ng (2008 ▶); Hassan *et al.* (2008 ▶).
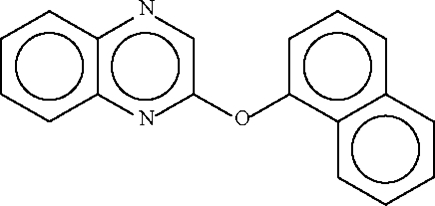


## Experimental

### 

#### Crystal data

C_18_H_12_N_2_O
*M* *_r_* = 272.30
Orthorhombic, 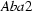

*a* = 18.2758 (6) Å
*b* = 18.5123 (6) Å
*c* = 7.7947 (3) Å
*V* = 2637.2 (2) Å^3^
*Z* = 8
Mo *K*α radiation
μ = 0.09 mm^−1^
*T* = 118 K
0.12 × 0.04 × 0.02 mm

#### Data collection

Bruker SMART APEX diffractometer
Absorption correction: none
12370 measured reflections
1626 independent reflections
1316 reflections with *I* > 2σ(*I*)
*R*_int_ = 0.071

#### Refinement

*R*[*F* ^2^ > 2σ(*F* ^2^)] = 0.038
*wR*(*F* ^2^) = 0.087
*S* = 1.02
1626 reflections
190 parameters
1 restraint
H-atom parameters constrained
Δρ_max_ = 0.19 e Å^−3^
Δρ_min_ = −0.24 e Å^−3^

### 

Data collection: *APEX2* (Bruker, 2007 ▶); cell refinement: *SAINT* (Bruker, 2007 ▶); data reduction: *SAINT*; program(s) used to solve structure: *SHELXS97* (Sheldrick, 2008 ▶); program(s) used to refine structure: *SHELXL97* (Sheldrick, 2008 ▶); molecular graphics: *X-SEED* (Barbour, 2001 ▶); software used to prepare material for publication: *publCIF* (Westrip, 2009 ▶).

## Supplementary Material

Crystal structure: contains datablocks global, I. DOI: 10.1107/S1600536809007867/tk2386sup1.cif

Structure factors: contains datablocks I. DOI: 10.1107/S1600536809007867/tk2386Isup2.hkl

Additional supplementary materials:  crystallographic information; 3D view; checkCIF report

## Figures and Tables

**Fig. 1.**
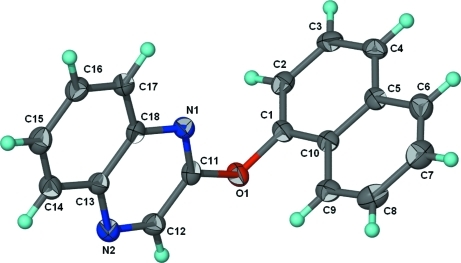
Thermal ellipsoid plot (Barbour, 2001) of the molecule of C18H12N2O at the 70% probability level. Hydrogen atoms are drawn as spheres of arbitrary radius.

## supplementary crystallographic information

### Experimental

1-Naphthol (2.88 g, 20 mmol) was mixed with sodium hydroxide (0.08 g, 20 mmol) in several drops of water. The water was then evaporated. The paste was heated with 2-chloroquinoxaline (3.29 g, 20 mmol) at 423–433 K for 6 h. The product was dissolved in water and the solution extracted with chloroform. The chloroform phase was dried over sodium sulfate; the evaporation of the solvent gave a product that was recrystallized from a chloroform/ether mixture.

### Refinement

Carbon-bound H-atoms were placed in calculated positions (C—H 0.95 Å) and were included in the refinement in the riding model approximation, with *U*(H) = 1.2*U*_eq_(C). In the absence of significant anomalous scattering effects, Friedel pairs were averaged in the final refinement.

### Crystal data

**Table:**

C_18_H_12_N_2_O	*F*(000) = 1136
*M**_r_* = 272.30	*D*_x_ = 1.372 Mg m^−^^3^
Orthorhombic, *A**b**a*2	Mo *K*α radiation, λ = 0.71073 Å
Hall symbol: A 2 -2ac	Cell parameters from 1495 reflections
*a* = 18.2758 (6) Å	θ = 2.2–21.2°
*b* = 18.5123 (6) Å	µ = 0.09 mm^−^^1^
*c* = 7.7947 (3) Å	*T* = 118 K
*V* = 2637.2 (2) Å^3^	Prism, colorless
*Z* = 8	0.12 × 0.04 × 0.02 mm

### Data collection

**Table:**

Bruker SMART APEX diffractometer	1316 reflections with *I* > 2σ(*I*)
Radiation source: fine-focus sealed tube	*R*_int_ = 0.071
graphite	θ_max_ = 27.5°, θ_min_ = 2.2°
ω scans	*h* = −22→23
12370 measured reflections	*k* = −24→23
1626 independent reflections	*l* = −10→10

### Refinement

**Table:**

Refinement on *F*^2^	Primary atom site location: structure-invariant direct methods
Least-squares matrix: full	Secondary atom site location: difference Fourier map
*R*[*F*^2^ > 2σ(*F*^2^)] = 0.038	Hydrogen site location: inferred from neighbouring sites
*wR*(*F*^2^) = 0.087	H-atom parameters constrained
*S* = 1.02	*w* = 1/[σ^2^(*F*_o_^2^) + (0.046*P*)^2^ + 0.5804*P*] where *P* = (*F*_o_^2^ + 2*F*_c_^2^)/3
1626 reflections	(Δ/σ)_max_ = 0.001
190 parameters	Δρ_max_ = 0.19 e Å^−^^3^
1 restraint	Δρ_min_ = −0.24 e Å^−^^3^

### Special details

**Table:**

Geometry. All e.s.d.'s (except the e.s.d. in the dihedral angle between two l.s. planes) are estimated using the full covariance matrix. The cell e.s.d.'s are taken into account individually in the estimation of e.s.d.'s in distances, angles and torsion angles; correlations between e.s.d.'s in cell parameters are only used when they are defined by crystal symmetry. An approximate (isotropic) treatment of cell e.s.d.'s is used for estimating e.s.d.'s involving l.s. planes.

### Fractional atomic coordinates and isotropic or equivalent isotropic displacement parameters (Å^2^)

**Table:**

	*x*	*y*	*z*	*U*_iso_*/*U*_eq_	
O1	0.19391 (9)	0.24366 (9)	0.5000 (2)	0.0217 (4)	
N1	0.15328 (11)	0.34927 (11)	0.3713 (3)	0.0192 (5)	
N2	0.30285 (11)	0.38838 (11)	0.3387 (3)	0.0218 (5)	
C1	0.12235 (13)	0.21546 (12)	0.4887 (4)	0.0192 (5)	
C2	0.07744 (14)	0.22066 (13)	0.6269 (4)	0.0222 (6)	
H2	0.0930	0.2454	0.7273	0.027*	
C3	0.00734 (14)	0.18873 (13)	0.6196 (3)	0.0234 (6)	
H3	−0.0246	0.1922	0.7155	0.028*	
C4	−0.01480 (14)	0.15306 (13)	0.4762 (3)	0.0231 (6)	
H4	−0.0619	0.1314	0.4737	0.028*	
C5	0.03121 (13)	0.14761 (12)	0.3301 (3)	0.0198 (5)	
C6	0.00887 (15)	0.11230 (14)	0.1784 (4)	0.0269 (6)	
H6	−0.0381	0.0903	0.1737	0.032*	
C7	0.05358 (16)	0.10925 (15)	0.0388 (4)	0.0301 (7)	
H7	0.0375	0.0857	−0.0627	0.036*	
C8	0.12373 (16)	0.14110 (15)	0.0448 (4)	0.0278 (6)	
H8	0.1546	0.1388	−0.0531	0.033*	
C9	0.14764 (14)	0.17502 (13)	0.1890 (4)	0.0219 (6)	
H9	0.1952	0.1957	0.1917	0.026*	
C10	0.10190 (13)	0.17973 (13)	0.3355 (3)	0.0180 (5)	
C11	0.20655 (14)	0.30943 (13)	0.4253 (3)	0.0189 (5)	
C12	0.28187 (14)	0.32836 (13)	0.4113 (3)	0.0213 (6)	
H12	0.3178	0.2964	0.4560	0.026*	
C13	0.24804 (15)	0.43279 (12)	0.2773 (3)	0.0190 (5)	
C14	0.26686 (14)	0.49812 (13)	0.1965 (4)	0.0232 (6)	
H14	0.3168	0.5114	0.1841	0.028*	
C15	0.21262 (15)	0.54275 (14)	0.1356 (4)	0.0247 (6)	
H15	0.2253	0.5866	0.0798	0.030*	
C16	0.13869 (15)	0.52397 (13)	0.1552 (4)	0.0231 (6)	
H16	0.1017	0.5552	0.1123	0.028*	
C17	0.11934 (14)	0.46108 (13)	0.2357 (3)	0.0212 (6)	
H17	0.0691	0.4494	0.2501	0.025*	
C18	0.17350 (13)	0.41378 (13)	0.2971 (3)	0.0190 (5)	

### Atomic displacement parameters (Å^2^)

**Table:**

	*U*^11^	*U*^22^	*U*^33^	*U*^12^	*U*^13^	*U*^23^
O1	0.0185 (9)	0.0192 (9)	0.0275 (10)	−0.0015 (7)	−0.0032 (8)	0.0043 (8)
N1	0.0177 (11)	0.0189 (10)	0.0211 (12)	−0.0005 (8)	−0.0005 (8)	0.0002 (9)
N2	0.0202 (11)	0.0228 (11)	0.0223 (11)	−0.0011 (8)	−0.0006 (10)	−0.0001 (10)
C1	0.0154 (13)	0.0156 (11)	0.0266 (14)	0.0005 (9)	−0.0035 (12)	0.0063 (11)
C2	0.0246 (14)	0.0183 (12)	0.0237 (14)	0.0038 (10)	−0.0013 (12)	0.0002 (11)
C3	0.0228 (14)	0.0215 (13)	0.0259 (15)	0.0046 (10)	0.0077 (12)	0.0030 (12)
C4	0.0164 (13)	0.0212 (12)	0.0316 (16)	0.0026 (9)	0.0011 (12)	0.0054 (12)
C5	0.0179 (13)	0.0159 (12)	0.0255 (14)	0.0015 (10)	−0.0038 (11)	0.0057 (11)
C6	0.0244 (14)	0.0236 (13)	0.0326 (16)	−0.0003 (11)	−0.0065 (13)	−0.0009 (13)
C7	0.0321 (16)	0.0316 (15)	0.0266 (16)	0.0045 (12)	−0.0094 (13)	−0.0040 (13)
C8	0.0325 (15)	0.0312 (15)	0.0198 (14)	0.0087 (12)	0.0021 (12)	0.0017 (12)
C9	0.0206 (13)	0.0194 (12)	0.0257 (14)	0.0031 (10)	0.0009 (11)	0.0065 (12)
C10	0.0184 (12)	0.0152 (11)	0.0205 (13)	0.0019 (9)	−0.0007 (11)	0.0045 (10)
C11	0.0225 (14)	0.0169 (12)	0.0172 (12)	−0.0021 (10)	−0.0020 (11)	−0.0011 (10)
C12	0.0193 (14)	0.0229 (13)	0.0217 (13)	0.0016 (10)	−0.0012 (11)	−0.0018 (11)
C13	0.0191 (12)	0.0193 (11)	0.0184 (13)	0.0000 (10)	−0.0003 (10)	−0.0023 (10)
C14	0.0226 (14)	0.0226 (12)	0.0243 (14)	−0.0037 (10)	0.0031 (12)	−0.0002 (12)
C15	0.0312 (15)	0.0203 (12)	0.0225 (14)	−0.0016 (11)	0.0019 (12)	0.0017 (11)
C16	0.0243 (14)	0.0199 (12)	0.0252 (14)	0.0032 (10)	−0.0034 (12)	−0.0006 (11)
C17	0.0192 (13)	0.0199 (12)	0.0245 (14)	−0.0004 (10)	−0.0005 (11)	−0.0023 (11)
C18	0.0205 (13)	0.0160 (11)	0.0204 (13)	−0.0010 (10)	0.0000 (11)	−0.0043 (11)

### Geometric parameters (Å, °)

**Table:**

O1—C11	1.369 (3)	C7—C8	1.412 (4)
O1—C1	1.411 (3)	C7—H7	0.9500
N1—C11	1.292 (3)	C8—C9	1.360 (4)
N1—C18	1.378 (3)	C8—H8	0.9500
N2—C12	1.304 (3)	C9—C10	1.418 (3)
N2—C13	1.382 (3)	C9—H9	0.9500
C1—C2	1.358 (4)	C11—C12	1.425 (3)
C1—C10	1.415 (3)	C12—H12	0.9500
C2—C3	1.412 (4)	C13—C14	1.406 (3)
C2—H2	0.9500	C13—C18	1.415 (4)
C3—C4	1.360 (4)	C14—C15	1.375 (4)
C3—H3	0.9500	C14—H14	0.9500
C4—C5	1.419 (4)	C15—C16	1.404 (4)
C4—H4	0.9500	C15—H15	0.9500
C5—C6	1.411 (4)	C16—C17	1.369 (4)
C5—C10	1.423 (3)	C16—H16	0.9500
C6—C7	1.362 (4)	C17—C18	1.405 (3)
C6—H6	0.9500	C17—H17	0.9500
			
C11—O1—C1	117.31 (18)	C10—C9—H9	119.8
C11—N1—C18	115.4 (2)	C1—C10—C9	123.5 (2)
C12—N2—C13	116.4 (2)	C1—C10—C5	117.4 (2)
C2—C1—O1	119.0 (2)	C9—C10—C5	119.1 (2)
C2—C1—C10	122.9 (2)	N1—C11—O1	121.3 (2)
O1—C1—C10	118.1 (2)	N1—C11—C12	124.2 (2)
C1—C2—C3	119.1 (2)	O1—C11—C12	114.5 (2)
C1—C2—H2	120.5	N2—C12—C11	121.8 (2)
C3—C2—H2	120.5	N2—C12—H12	119.1
C4—C3—C2	120.4 (2)	C11—C12—H12	119.1
C4—C3—H3	119.8	N2—C13—C14	119.3 (2)
C2—C3—H3	119.8	N2—C13—C18	120.8 (2)
C3—C4—C5	121.2 (2)	C14—C13—C18	119.9 (2)
C3—C4—H4	119.4	C15—C14—C13	119.7 (2)
C5—C4—H4	119.4	C15—C14—H14	120.2
C6—C5—C4	122.3 (2)	C13—C14—H14	120.2
C6—C5—C10	118.8 (2)	C14—C15—C16	120.5 (2)
C4—C5—C10	119.0 (2)	C14—C15—H15	119.7
C7—C6—C5	121.0 (2)	C16—C15—H15	119.7
C7—C6—H6	119.5	C17—C16—C15	120.6 (2)
C5—C6—H6	119.5	C17—C16—H16	119.7
C6—C7—C8	120.0 (3)	C15—C16—H16	119.7
C6—C7—H7	120.0	C16—C17—C18	120.3 (2)
C8—C7—H7	120.0	C16—C17—H17	119.9
C9—C8—C7	120.8 (3)	C18—C17—H17	119.9
C9—C8—H8	119.6	N1—C18—C17	119.6 (2)
C7—C8—H8	119.6	N1—C18—C13	121.3 (2)
C8—C9—C10	120.3 (2)	C17—C18—C13	119.1 (2)
C8—C9—H9	119.8		
			
C11—O1—C1—C2	−101.4 (3)	C18—N1—C11—O1	−179.3 (2)
C11—O1—C1—C10	81.6 (3)	C18—N1—C11—C12	−0.3 (4)
O1—C1—C2—C3	−176.3 (2)	C1—O1—C11—N1	10.9 (3)
C10—C1—C2—C3	0.5 (3)	C1—O1—C11—C12	−168.2 (2)
C1—C2—C3—C4	0.2 (3)	C13—N2—C12—C11	0.4 (4)
C2—C3—C4—C5	−0.7 (4)	N1—C11—C12—N2	−1.0 (4)
C3—C4—C5—C6	−178.6 (2)	O1—C11—C12—N2	178.1 (2)
C3—C4—C5—C10	0.5 (3)	C12—N2—C13—C14	−179.6 (2)
C4—C5—C6—C7	178.6 (2)	C12—N2—C13—C18	1.4 (4)
C10—C5—C6—C7	−0.5 (4)	N2—C13—C14—C15	−179.7 (2)
C5—C6—C7—C8	0.6 (4)	C18—C13—C14—C15	−0.7 (4)
C6—C7—C8—C9	0.1 (4)	C13—C14—C15—C16	0.7 (4)
C7—C8—C9—C10	−0.8 (4)	C14—C15—C16—C17	0.2 (4)
C2—C1—C10—C9	178.8 (2)	C15—C16—C17—C18	−1.2 (4)
O1—C1—C10—C9	−4.4 (3)	C11—N1—C18—C17	−179.5 (2)
C2—C1—C10—C5	−0.7 (3)	C11—N1—C18—C13	2.1 (3)
O1—C1—C10—C5	176.1 (2)	C16—C17—C18—N1	−177.3 (2)
C8—C9—C10—C1	−178.6 (2)	C16—C17—C18—C13	1.2 (4)
C8—C9—C10—C5	0.9 (4)	N2—C13—C18—N1	−2.8 (4)
C6—C5—C10—C1	179.3 (2)	C14—C13—C18—N1	178.2 (2)
C4—C5—C10—C1	0.2 (3)	N2—C13—C18—C17	178.8 (2)
C6—C5—C10—C9	−0.2 (3)	C14—C13—C18—C17	−0.3 (4)
C4—C5—C10—C9	−179.3 (2)		
